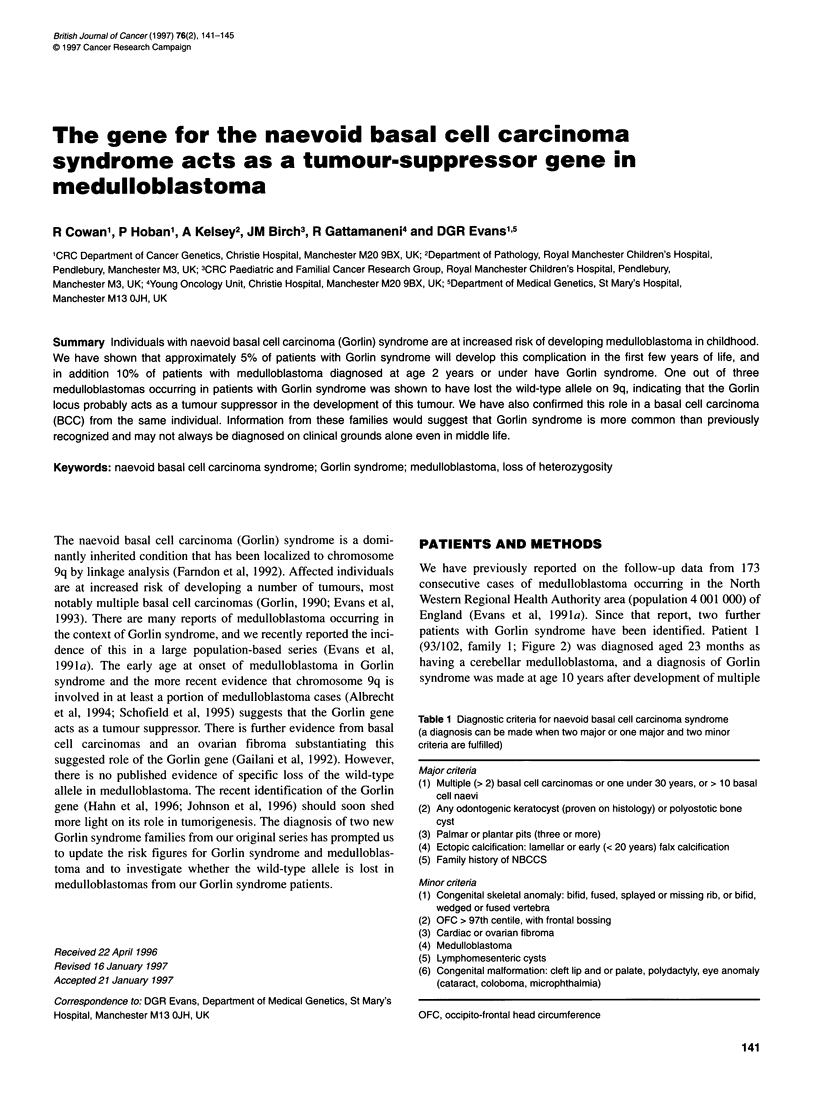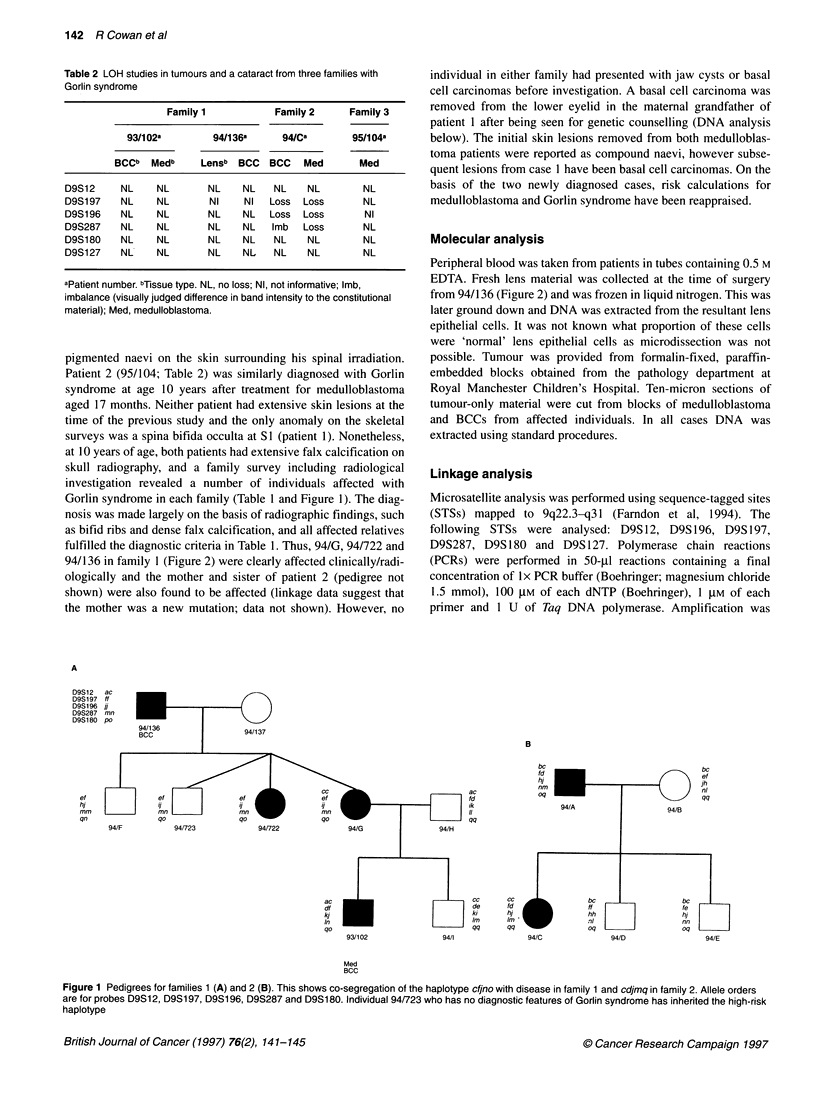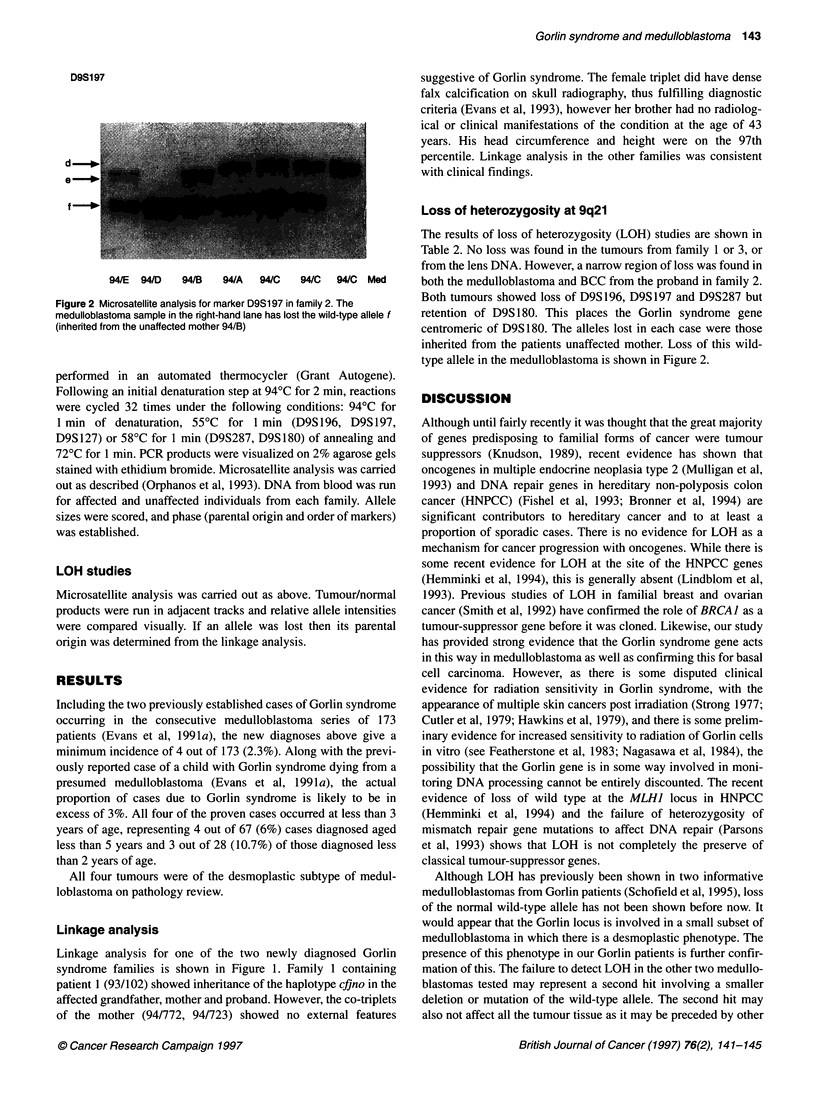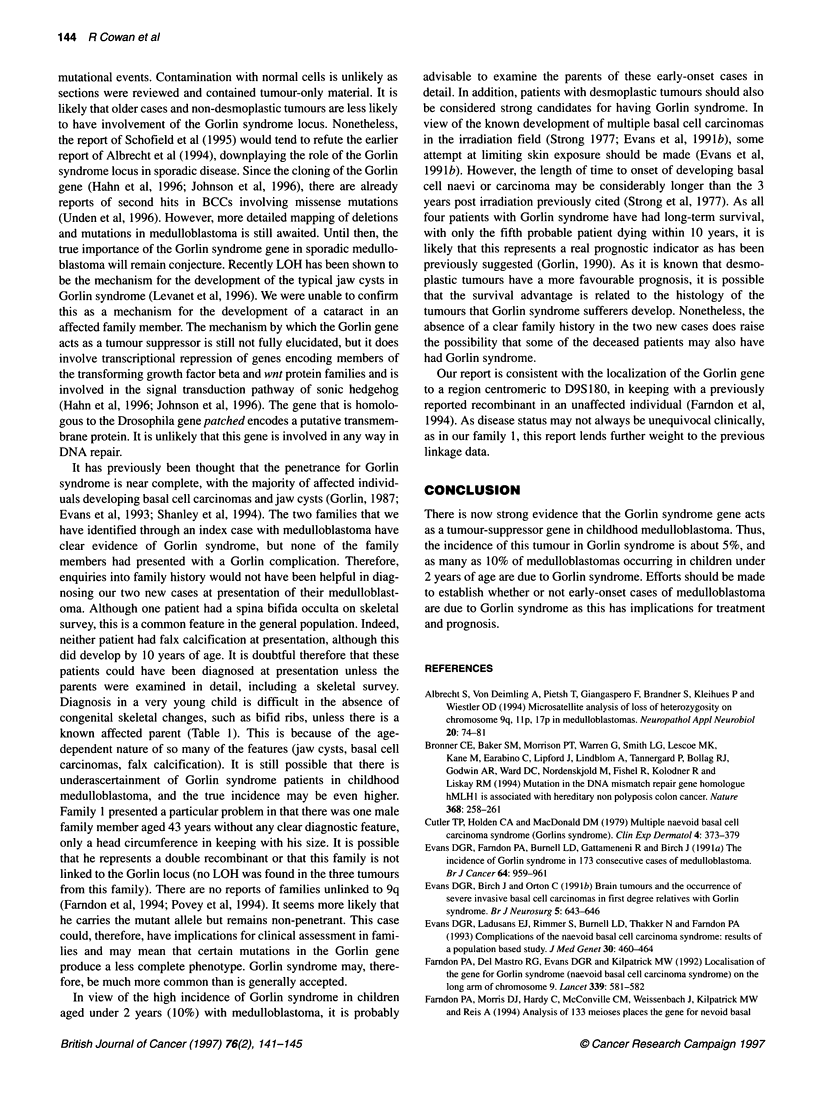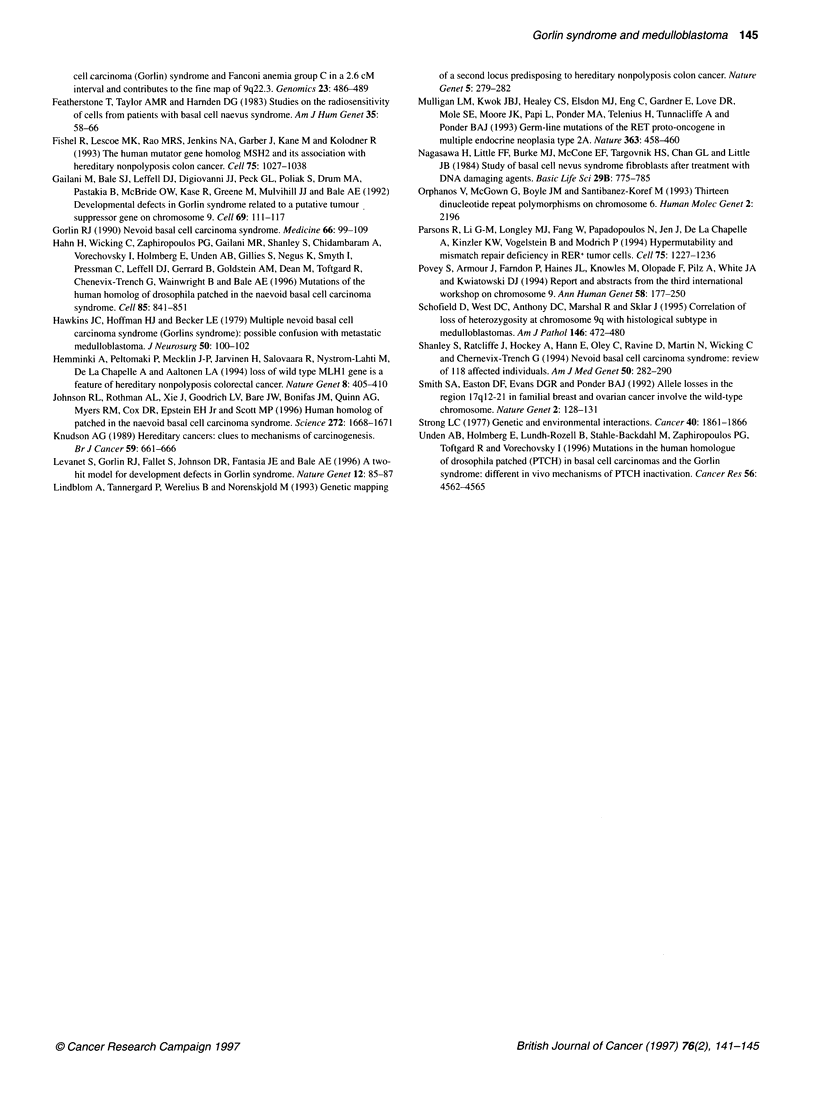# The gene for the naevoid basal cell carcinoma syndrome acts as a tumour-suppressor gene in medulloblastoma.

**DOI:** 10.1038/bjc.1997.354

**Published:** 1997

**Authors:** R. Cowan, P. Hoban, A. Kelsey, J. M. Birch, R. Gattamaneni, D. G. Evans

**Affiliations:** CRC Department of Cancer Genetics, Christie Hospital, Manchester, UK.

## Abstract

**Images:**


British Journal of Cancer (1997) 76(2), 141–145
© 1997 Cancer Research Campaign

### The gene for the naevoid basal cell carcinoma syndrome acts as a tumour-suppressor gene in medulloblastoma

R Cowan[1], P Hoban[1], A Kelsey[2], JM Birch[3], R Gattamaneni[4] and DGR Evans[1,5]

[1]CRC Department of Cancer Genetics, Christie Hospital, Manchester M20 9BX, UK; [2]Department of Pathology, Royal Manchester Children's Hospital, Pendlebury, Manchester M3, UK; [3]CRC Paediatric and Familial Cancer Research Group, Royal Manchester Children's Hospital, Pendlebury, Manchester M3, UK; [4]Young Oncology Unit, Christie Hospital, Manchester M20 9BX, UK; [5]Department of Medical Genetics, St Mary's Hospital, Manchester M13 0JH, UK

**Summary** Individuals with naevoid basal cell carcinoma (Gorlin) syndrome are at increased risk of developing medulloblastoma in childhood. We have shown that approximately 5% of patients with Gorlin syndrome will develop this complication in the first few years of life, and in addition 10% of patients with medulloblastoma diagnosed at age 2 years or under have Gorlin syndrome. One out of three medulloblastomas occurring in patients with Gorlin syndrome was shown to have lost the wild-type allele on 9q, indicating that the Gorlin locus probably acts as a tumour suppressor in the development of this tumour. We have also confirmed this role in a basal cell carcinoma (BCC) from the same individual. Information from these families would suggest that Gorlin syndrome is more common than previously recognized and may not always be diagnosed on clinical grounds alone even in middle life.

**Keywords:** naevoid basal cell carcinoma syndrome; Gorlin syndrome; medulloblastoma, loss of heterozygosity

The naevoid basal cell carcinoma (Gorlin) syndrome is a dominantly inherited condition that has been localized to chromosome 9q by linkage analysis (Farndon et al, 1992). Affected individuals are at increased risk of developing a number of tumours, most notably multiple basal cell carcinomas (Gorlin, 1990; Evans et al, 1993). There are many reports of medulloblastoma occurring in the context of Gorlin syndrome, and we recently reported the incidence of this in a large population-based series (Evans et al, 1991*a*). The early age at onset of medulloblastoma in Gorlin syndrome and the more recent evidence that chromosome 9q is involved in at least a portion of medulloblastoma cases (Albrecht et al, 1994; Schofield et al, 1995) suggests that the Gorlin gene acts as a tumour suppressor. There is further evidence from basal cell carcinomas and an ovarian fibroma substantiating this suggested role of the Gorlin gene (Gailani et al, 1992). However, there is no published evidence of specific loss of the wild-type allele in medulloblastoma. The recent identification of the Gorlin gene (Hahn et al, 1996; Johnson et al, 1996) should soon shed more light on its role in tumorigenesis. The diagnosis of two new Gorlin syndrome families from our original series has prompted us to update the risk figures for Gorlin syndrome and medulloblastoma and to investigate whether the wild-type allele is lost in medulloblastomas from our Gorlin syndrome patients.

*Received 22 April 1996*
*Revised 16 January 1997*
*Accepted 21 January 1997*

*Correspondence to:* DGR Evans, Department of Medical Genetics, St Mary's Hospital, Manchester M13 0JH, UK

### PATIENTS AND METHODS

We have previously reported on the follow-up data from 173 consecutive cases of medulloblastoma occurring in the North Western Regional Health Authority area (population 4 001 000) of England (Evans et al, 1991*a*). Since that report, two further patients with Gorlin syndrome have been identified. Patient 1 (93/102, family 1; Figure 2) was diagnosed aged 23 months as having a cerebellar medulloblastoma, and a diagnosis of Gorlin syndrome was made at age 10 years after development of multiple

**Table 1** Diagnostic criteria for naevoid basal cell carcinoma syndrome (a diagnosis can be made when two major or one major and two minor criteria are fulfilled)

*Major criteria*
(1) Multiple (> 2) basal cell carcinomas or one under 30 years, or > 10 basal cell naevi
(2) Any odontogenic keratocyst (proven on histology) or polyostotic bone cyst
(3) Palmar or plantar pits (three or more)
(4) Ectopic calcification: lamellar or early (< 20 years) falx calcification
(5) Family history of NBCCS

*Minor criteria*
(1) Congenital skeletal anomaly: bifid, fused, splayed or missing rib, or bifid, wedged or fused vertebra
(2) OFC > 97th centile, with frontal bossing
(3) Cardiac or ovarian fibroma
(4) Medulloblastoma
(5) Lymphomesenteric cysts
(6) Congenital malformation: cleft lip and or palate, polydactyly, eye anomaly (cataract, coloboma, microphthalmia)

OFC, occipito-frontal head circumference

**Table 2** LOH studies in tumours and a cataract from three families with Gorlin syndrome

| | Family 1 | | | | Family 2 | | Family 3 |
|---|---|---|---|---|---|---|---|
| | 93/102[a] | | 94/136[a] | | 94/C[a] | | 95/104[a] |
| | BCC[b] | Med[b] | Lens[b] | BCC | BCC | Med | Med |
| D9S12 | NL | NL | NL | NL | NL | NL | NL |
| D9S197 | NL | NL | NI | NI | Loss | Loss | NL |
| D9S196 | NL | NL | NL | NL | Loss | Loss | NI |
| D9S287 | NL | NL | NL | NL | Imb | Loss | NL |
| D9S180 | NL | NL | NL | NL | NL | NL | NL |
| D9S127 | NL | NL | NL | NL | NL | NL | NL |

[a]Patient number. [b]Tissue type. NL, no loss; NI, not informative; Imb, imbalance (visually judged difference in band intensity to the constitutional material); Med, medulloblastoma.

pigmented naevi on the skin surrounding his spinal irradiation. Patient 2 (95/104; Table 2) was similarly diagnosed with Gorlin syndrome at age 10 years after treatment for medulloblastoma aged 17 months. Neither patient had extensive skin lesions at the time of the previous study and the only anomaly on the skeletal surveys was a spina bifida occulta at S1 (patient 1). Nonetheless, at 10 years of age, both patients had extensive falx calcification on skull radiography, and a family survey including radiological investigation revealed a number of individuals affected with Gorlin syndrome in each family (Table 1 and Figure 1). The diagnosis was made largely on the basis of radiographic findings, such as bifid ribs and dense falx calcification, and all affected relatives fulfilled the diagnostic criteria in Table 1. Thus, 94/G, 94/722 and 94/136 in family 1 (Figure 2) were clearly affected clinically/radiologically and the mother and sister of patient 2 (pedigree not shown) were also found to be affected (linkage data suggest that the mother was a new mutation; data not shown). However, no individual in either family had presented with jaw cysts or basal cell carcinomas before investigation. A basal cell carcinoma was removed from the lower eyelid in the maternal grandfather of patient 1 after being seen for genetic counselling (DNA analysis below). The initial skin lesions removed from both medulloblastoma patients were reported as compound naevi, however subsequent lesions from case 1 have been basal cell carcinomas. On the basis of the two newly diagnosed cases, risk calculations for medulloblastoma and Gorlin syndrome have been reappraised.

### Molecular analysis

Peripheral blood was taken from patients in tubes containing 0.5 M EDTA. Fresh lens material was collected at the time of surgery from 94/136 (Figure 2) and was frozen in liquid nitrogen. This was later ground down and DNA was extracted from the resultant lens epithelial cells. It was not known what proportion of these cells were 'normal' lens epithelial cells as microdissection was not possible. Tumour was provided from formalin-fixed, paraffin-embedded blocks obtained from the pathology department at Royal Manchester Children's Hospital. Ten-micron sections of tumour-only material were cut from blocks of medulloblastoma and BCCs from affected individuals. In all cases DNA was extracted using standard procedures.

### Linkage analysis

Microsatellite analysis was performed using sequence-tagged sites (STSs) mapped to 9q22.3–q31 (Farndon et al, 1994). The following STSs were analysed: D9S12, D9S196, D9S197, D9S287, D9S180 and D9S127. Polymerase chain reactions (PCRs) were performed in 50-μl reactions containing a final concentration of 1× PCR buffer (Boehringer; magnesium chloride 1.5 mmol), 100 μM of each dNTP (Boehringer), 1 μM of each primer and 1 U of *Taq* DNA polymerase. Amplification was

**Figure 1** Pedigrees for families 1 (**A**) and 2 (**B**). This shows co-segregation of the haplotype *cfjno* with disease in family 1 and *cdjmq* in family 2. Allele orders are for probes D9S12, D9S197, D9S196, D9S287 and D9S180. Individual 94/723 who has no diagnostic features of Gorlin syndrome has inherited the high-risk haplotype

© Cancer Research Campaign 1997

**Figure 2** Microsatellite analysis for marker D9S197 in family 2. The medulloblastoma sample in the right-hand lane has lost the wild-type allele *f* (inherited from the unaffected mother 94/B)

performed in an automated thermocycler (Grant Autogene). Following an initial denaturation step at 94°C for 2 min, reactions were cycled 32 times under the following conditions: 94°C for 1 min of denaturation, 55°C for 1 min (D9S196, D9S197, D9S127) or 58°C for 1 min (D9S287, D9S180) of annealing and 72°C for 1 min. PCR products were visualized on 2% agarose gels stained with ethidium bromide. Microsatellite analysis was carried out as described (Orphanos et al, 1993). DNA from blood was run for affected and unaffected individuals from each family. Allele sizes were scored, and phase (parental origin and order of markers) was established.

### LOH studies

Microsatellite analysis was carried out as above. Tumour/normal products were run in adjacent tracks and relative allele intensities were compared visually. If an allele was lost then its parental origin was determined from the linkage analysis.

### RESULTS

Including the two previously established cases of Gorlin syndrome occurring in the consecutive medulloblastoma series of 173 patients (Evans et al, 1991*a*), the new diagnoses above give a minimum incidence of 4 out of 173 (2.3%). Along with the previously reported case of a child with Gorlin syndrome dying from a presumed medulloblastoma (Evans et al, 1991*a*), the actual proportion of cases due to Gorlin syndrome is likely to be in excess of 3%. All four of the proven cases occurred at less than 3 years of age, representing 4 out of 67 (6%) cases diagnosed aged less than 5 years and 3 out of 28 (10.7%) of those diagnosed less than 2 years of age.

All four tumours were of the desmoplastic subtype of medulloblastoma on pathology review.

### Linkage analysis

Linkage analysis for one of the two newly diagnosed Gorlin syndrome families is shown in Figure 1. Family 1 containing patient 1 (93/102) showed inheritance of the haplotype *cfjno* in the affected grandfather, mother and proband. However, the co-triplets of the mother (94/772, 94/723) showed no external features suggestive of Gorlin syndrome. The female triplet did have dense falx calcification on skull radiography, thus fulfilling diagnostic criteria (Evans et al, 1993), however her brother had no radiological or clinical manifestations of the condition at the age of 43 years. His head circumference and height were on the 97th percentile. Linkage analysis in the other families was consistent with clinical findings.

### Loss of heterozygosity at 9q21

The results of loss of heterozygosity (LOH) studies are shown in Table 2. No loss was found in the tumours from family 1 or 3, or from the lens DNA. However, a narrow region of loss was found in both the medulloblastoma and BCC from the proband in family 2. Both tumours showed loss of D9S196, D9S197 and D9S287 but retention of D9S180. This places the Gorlin syndrome gene centromeric of D9S180. The alleles lost in each case were those inherited from the patients unaffected mother. Loss of this wild-type allele in the medulloblastoma is shown in Figure 2.

### DISCUSSION

Although until fairly recently it was thought that the great majority of genes predisposing to familial forms of cancer were tumour suppressors (Knudson, 1989), recent evidence has shown that oncogenes in multiple endocrine neoplasia type 2 (Mulligan et al, 1993) and DNA repair genes in hereditary non-polyposis colon cancer (HNPCC) (Fishel et al, 1993; Bronner et al, 1994) are significant contributors to hereditary cancer and to at least a proportion of sporadic cases. There is no evidence for LOH as a mechanism for cancer progression with oncogenes. While there is some recent evidence for LOH at the site of the HNPCC genes (Hemminki et al, 1994), this is generally absent (Lindblom et al, 1993). Previous studies of LOH in familial breast and ovarian cancer (Smith et al, 1992) have confirmed the role of *BRCA1* as a tumour-suppressor gene before it was cloned. Likewise, our study has provided strong evidence that the Gorlin syndrome gene acts in this way in medulloblastoma as well as confirming this for basal cell carcinoma. However, as there is some disputed clinical evidence for radiation sensitivity in Gorlin syndrome, with the appearance of multiple skin cancers post irradiation (Strong 1977; Cutler et al, 1979; Hawkins et al, 1979), and there is some preliminary evidence for increased sensitivity to radiation of Gorlin cells in vitro (see Featherstone et al, 1983; Nagasawa et al, 1984), the possibility that the Gorlin gene is in some way involved in monitoring DNA processing cannot be entirely discounted. The recent evidence of loss of wild type at the *MLH1* locus in HNPCC (Hemminki et al, 1994) and the failure of heterozygosity of mismatch repair gene mutations to affect DNA repair (Parsons et al, 1993) shows that LOH is not completely the preserve of classical tumour-suppressor genes.

Although LOH has previously been shown in two informative medulloblastomas from Gorlin patients (Schofield et al, 1995), loss of the normal wild-type allele has not been shown before now. It would appear that the Gorlin locus is involved in a small subset of medulloblastoma in which there is a desmoplastic phenotype. The presence of this phenotype in our Gorlin patients is further confirmation of this. The failure to detect LOH in the other two medulloblastomas tested may represent a second hit involving a smaller deletion or mutation of the wild-type allele. The second hit may also not affect all the tumour tissue as it may be preceded by other

© Cancer Research Campaign 1997 

mutational events. Contamination with normal cells is unlikely as sections were reviewed and contained tumour-only material. It is likely that older cases and non-desmoplastic tumours are less likely to have involvement of the Gorlin syndrome locus. Nonetheless, the report of Schofield et al (1995) would tend to refute the earlier report of Albrecht et al (1994), downplaying the role of the Gorlin syndrome locus in sporadic disease. Since the cloning of the Gorlin gene (Hahn et al, 1996; Johnson et al, 1996), there are already reports of second hits in BCCs involving missense mutations (Unden et al, 1996). However, more detailed mapping of deletions and mutations in medulloblastoma is still awaited. Until then, the true importance of the Gorlin syndrome gene in sporadic medulloblastoma will remain conjecture. Recently LOH has been shown to be the mechanism for the development of the typical jaw cysts in Gorlin syndrome (Levanet et al, 1996). We were unable to confirm this as a mechanism for the development of a cataract in an affected family member. The mechanism by which the Gorlin gene acts as a tumour suppressor is still not fully elucidated, but it does involve transcriptional repression of genes encoding members of the transforming growth factor beta and *wnt* protein families and is involved in the signal transduction pathway of sonic hedgehog (Hahn et al, 1996; Johnson et al, 1996). The gene that is homologous to the Drosophila gene *patched* encodes a putative transmembrane protein. It is unlikely that this gene is involved in any way in DNA repair.

It has previously been thought that the penetrance for Gorlin syndrome is near complete, with the majority of affected individuals developing basal cell carcinomas and jaw cysts (Gorlin, 1987; Evans et al, 1993; Shanley et al, 1994). The two families that we have identified through an index case with medulloblastoma have clear evidence of Gorlin syndrome, but none of the family members had presented with a Gorlin complication. Therefore, enquiries into family history would not have been helpful in diagnosing our two new cases at presentation of their medulloblastoma. Although one patient had a spina bifida occulta on skeletal survey, this is a common feature in the general population. Indeed, neither patient had falx calcification at presentation, although this did develop by 10 years of age. It is doubtful therefore that these patients could have been diagnosed at presentation unless the parents were examined in detail, including a skeletal survey. Diagnosis in a very young child is difficult in the absence of congenital skeletal changes, such as bifid ribs, unless there is a known affected parent (Table 1). This is because of the age-dependent nature of so many of the features (jaw cysts, basal cell carcinomas, falx calcification). It is still possible that there is underascertainment of Gorlin syndrome patients in childhood medulloblastoma, and the true incidence may be even higher. Family 1 presented a particular problem in that there was one male family member aged 43 years without any clear diagnostic feature, only a head circumference in keeping with his size. It is possible that he represents a double recombinant or that this family is not linked to the Gorlin locus (no LOH was found in the three tumours from this family). There are no reports of families unlinked to 9q (Farndon et al, 1994; Povey et al, 1994). It seems more likely that he carries the mutant allele but remains non-penetrant. This case could, therefore, have implications for clinical assessment in families and may mean that certain mutations in the Gorlin gene produce a less complete phenotype. Gorlin syndrome may, therefore, be much more common than is generally accepted.

In view of the high incidence of Gorlin syndrome in children aged under 2 years (10%) with medulloblastoma, it is probably advisable to examine the parents of these early-onset cases in detail. In addition, patients with desmoplastic tumours should also be considered strong candidates for having Gorlin syndrome. In view of the known development of multiple basal cell carcinomas in the irradiation field (Strong 1977; Evans et al, 1991*b*), some attempt at limiting skin exposure should be made (Evans et al, 1991*b*). However, the length of time to onset of developing basal cell naevi or carcinoma may be considerably longer than the 3 years post irradiation previously cited (Strong et al, 1977). As all four patients with Gorlin syndrome have had long-term survival, with only the fifth probable patient dying within 10 years, it is likely that this represents a real prognostic indicator as has been previously suggested (Gorlin, 1990). As it is known that desmoplastic tumours have a more favourable prognosis, it is possible that the survival advantage is related to the histology of the tumours that Gorlin syndrome sufferers develop. Nonetheless, the absence of a clear family history in the two new cases does raise the possibility that some of the deceased patients may also have had Gorlin syndrome.

Our report is consistent with the localization of the Gorlin gene to a region centromeric to D9S180, in keeping with a previously reported recombinant in an unaffected individual (Farndon et al, 1994). As disease status may not always be unequivocal clinically, as in our family 1, this report lends further weight to the previous linkage data.

### CONCLUSION

There is now strong evidence that the Gorlin syndrome gene acts as a tumour-suppressor gene in childhood medulloblastoma. Thus, the incidence of this tumour in Gorlin syndrome is about 5%, and as many as 10% of medulloblastomas occurring in children under 2 years of age are due to Gorlin syndrome. Efforts should be made to establish whether or not early-onset cases of medulloblastoma are due to Gorlin syndrome as this has implications for treatment and prognosis.

### REFERENCES

Albrecht S, Von Deimling A, Pietsh T, Giangaspero F, Brandner S, Kleihues P and Wiestler OD (1994) Microsatellite analysis of loss of heterozygosity on chromosome 9q, 11p, 17p in medulloblastomas. *Neuropathol Appl Neurobiol* **20**: 74–81

Bronner CE, Baker SM, Morrison PT, Warren G, Smith LG, Lescoe MK, Kane M, Earabino C, Lipford J, Lindblom A, Tannergard P, Bollag RJ, Godwin AR, Ward DC, Nordenskjold M, Fishel R, Kolodner R and Liskay RM (1994) Mutation in the DNA mismatch repair gene homologue hMLH1 is associated with hereditary non polyposis colon cancer. *Nature* **368**: 258–261

Cutler TP, Holden CA and MacDonald DM (1979) Multiple naevoid basal cell carcinoma syndrome (Gorlins syndrome). *Clin Exp Dermatol* **4**: 373–379

Evans DGR, Farndon PA, Burnell LD, Gattameneni R and Birch J (1991*a*) The incidence of Gorlin syndrome in 173 consecutive cases of medulloblastoma. *Br J Cancer* **64**: 959–961

Evans DGR, Birch J and Orton C (1991*b*) Brain tumours and the occurrence of severe invasive basal cell carcinomas in first degree relatives with Gorlin syndrome. *Br J Neurosurg* **5**: 643–646

Evans DGR, Ladusans EJ, Rimmer S, Burnell LD, Thakker N and Farndon PA (1993) Complications of the naevoid basal cell carcinoma syndrome: results of a population based study. *J Med Genet* **30**: 460–464

Farndon PA, Del Mastro RG, Evans DGR and Kilpatrick MW (1992) Localisation of the gene for Gorlin syndrome (naevoid basal cell carcinoma syndrome) on the long arm of chromosome 9. *Lancet* **339**: 581–582

Farndon PA, Morris DJ, Hardy C, McConville CM, Weissenbach J, Kilpatrick MW and Reis A (1994) Analysis of 133 meioses places the gene for nevoid basal

 © Cancer Research Campaign 1997

cell carcinoma (Gorlin) syndrome and Fanconi anemia group C in a 2.6 cM interval and contributes to the fine map of 9q22.3. *Genomics* **23**: 486–489

Featherstone T, Taylor AMR and Harnden DG (1983) Studies on the radiosensitivity of cells from patients with basal cell naevus syndrome. *Am J Hum Genet* **35**: 58–66

Fishel R, Lescoe MK, Rao MRS, Jenkins NA, Garber J, Kane M and Kolodner R (1993) The human mutator gene homolog MSH2 and its association with hereditary nonpolyposis colon cancer. *Cell* **75**: 1027–1038

Gailani M, Bale SJ, Leffell DJ, Digiovanni JJ, Peck GL, Poliak S, Drum MA, Pastakia B, McBride OW, Kase R, Greene M, Mulvihill JJ and Bale AE (1992) Developmental defects in Gorlin syndrome related to a putative tumour suppressor gene on chromosome 9. *Cell* **69**: 111–117

Gorlin RJ (1990) Nevoid basal cell carcinoma syndrome. *Medicine* **66**: 99–109

Hahn H, Wicking C, Zaphiropoulos PG, Gailani MR, Shanley S, Chidambaram A, Vorechovsky I, Holmberg E, Unden AB, Gillies S, Negus K, Smyth I, Pressman C, Leffell DJ, Gerrard B, Goldstein AM, Dean M, Toftgard R, Chenevix-Trench G, Wainwright B and Bale AE (1996) Mutations of the human homolog of drosophila patched in the naevoid basal cell carcinoma syndrome. *Cell* **85**: 841–851

Hawkins JC, Hoffman HJ and Becker LE (1979) Multiple nevoid basal cell carcinoma syndrome (Gorlins syndrome): possible confusion with metastatic medulloblastoma. *J Neurosurg* **50**: 100–102

Hemminki A, Peltomaki P, Mecklin J-P, Jarvinen H, Salovaara R, Nystrom-Lahti M, De La Chapelle A and Aaltonen LA (1994) loss of wild type MLH1 gene is a feature of hereditary nonpolyposis colorectal cancer. *Nature Genet* **8**: 405–410

Johnson RL, Rothman AL, Xie J, Goodrich LV, Bare JW, Bonifas JM, Quinn AG, Myers RM, Cox DR, Epstein EH Jr and Scott MP (1996) Human homolog of patched in the naevoid basal cell carcinoma syndrome. *Science* **272**: 1668–1671

Knudson AG (1989) Hereditary cancers: clues to mechanisms of carcinogenesis. *Br J Cancer* **59**: 661–666

Levanet S, Gorlin RJ, Fallet S, Johnson DR, Fantasia JE and Bale AE (1996) A two-hit model for development defects in Gorlin syndrome. *Nature Genet* **12**: 85–87

Lindblom A, Tannergard P, Werelius B and Norenskjold M (1993) Genetic mapping of a second locus predisposing to hereditary nonpolyposis colon cancer. *Nature Genet* **5**: 279–282

Mulligan LM, Kwok JBJ, Healey CS, Elsdon MJ, Eng C, Gardner E, Love DR, Mole SE, Moore JK, Papi L, Ponder MA, Telenius H, Tunnacliffe A and Ponder BAJ (1993) Germ-line mutations of the RET proto-oncogene in multiple endocrine neoplasia type 2A. *Nature* **363**: 458–460

Nagasawa H, Little FF, Burke MJ, McCone EF, Targovnik HS, Chan GL and Little JB (1984) Study of basal cell nevus syndrome fibroblasts after treatment with DNA damaging agents. *Basic Life Sci* **29B**: 775–785

Orphanos V, McGown G, Boyle JM and Santibanez-Koref M (1993) Thirteen dinucleotide repeat polymorphisms on chromosome 6. *Human Molec Genet* **2**: 2196

Parsons R, Li G-M, Longley MJ, Fang W, Papadopoulos N, Jen J, De La Chapelle A, Kinzler KW, Vogelstein B and Modrich P (1994) Hypermutability and mismatch repair deficiency in RER$^+$ tumor cells. *Cell* **75**: 1227–1236

Povey S, Armour J, Farndon P, Haines JL, Knowles M, Olopade F, Pilz A, White JA and Kwiatowski DJ (1994) Report and abstracts from the third international workshop on chromosome 9. *Ann Human Genet* **58**: 177–250

Schofield D, West DC, Anthony DC, Marshal R and Sklar J (1995) Correlation of loss of heterozygosity at chromosome 9q with histological subtype in medulloblastomas. *Am J Pathol* **146**: 472–480

Shanley S, Ratcliffe J, Hockey A, Hann E, Oley C, Ravine D, Martin N, Wicking C and Chernevix-Trench G (1994) Nevoid basal cell carcinoma syndrome: review of 118 affected individuals. *Am J Med Genet* **50**: 282–290

Smith SA, Easton DF, Evans DGR and Ponder BAJ (1992) Allele losses in the region 17q12-21 in familial breast and ovarian cancer involve the wild-type chromosome. *Nature Genet* **2**: 128–131

Strong LC (1977) Genetic and environmental interactions. *Cancer* **40**: 1861–1866

Unden AB, Holmberg E, Lundh-Rozell B, Stahle-Backdahl M, Zaphiropoulos PG, Toftgard R and Vorechovsky I (1996) Mutations in the human homologue of drosophila patched (PTCH) in basal cell carcinomas and the Gorlin syndrome: different in vivo mechanisms of PTCH inactivation. *Cancer Res* **56**: 4562–4565

© Cancer Research Campaign 1997